# Supplementary material for: MetaRibo-Seq measures translation in microbiomes
Source: Nat Commun. 2020 Jun 29;11:3268. doi: 10.1038/s41467-020-17081-z (PMC7324362; doi:10.1038/s41467-020-17081-z)
Supplement: Supplementary file 10 — Supplementary Data 7 [file 41467_2020_17081_MOESM10_ESM.zip › File2/Confidence_High_Taxonomy/425524_out.krona.html]

Javascript must be enabled to view this page.

members
magnitude
magnitudeUnassigned
count
unassigned
taxon
rank

425524\_out

220

2
220
superkingdom

976
phylum
220

class
220
200643

order
220
1
171549

SRS046712\_contig\_number\_556

2005525
186
family

375288

SRS011239\_contig\_number\_26302SRS015663\_contig\_number\_23658SRS019968\_contig\_number\_17815SRS043667\_contig\_number\_contig-100\_684.46249SRS045244\_contig\_number\_contig-100\_510.67174SRS048060\_contig\_number\_3669SRS048870\_contig\_number\_22637SRS056519\_contig\_number\_17742SRS075878\_contig\_number\_13341SRS077294\_contig\_number\_11574SRS077335\_contig\_number\_8799SRS103987\_contig\_number\_contig-100\_989.186806SRS1054928\_contig\_number\_2171SRS148970\_contig\_number\_814
186
genus
14


SRS011134\_contig\_number\_39722SRS012273\_contig\_number\_33348SRS012969\_contig\_number\_13087SRS013098\_contig\_number\_42357SRS013638\_contig\_number\_contig-100\_129.89320SRS014235\_contig\_number\_37104SRS014287\_contig\_number\_13510SRS014313\_contig\_number\_2126SRS014412\_contig\_number\_17615SRS014948\_contig\_number\_22140SRS014979\_contig\_number\_20174SRS015065\_contig\_number\_25918SRS015217\_contig\_number\_23734SRS015264\_contig\_number\_12349SRS015579\_contig\_number\_15873SRS015794\_contig\_number\_10023SRS015960\_contig\_number\_7331SRS016056\_contig\_number\_2145SRS016095\_contig\_number\_3124SRS016267\_contig\_number\_7695SRS016989\_contig\_number\_10663SRS017191\_contig\_number\_19483SRS018313\_contig\_number\_contig-100\_16.80150SRS018351\_contig\_number\_7053SRS018541\_contig\_number\_2012SRS018575\_contig\_number\_706SRS018623\_contig\_number\_18020SRS018656\_contig\_number\_8747SRS018836\_contig\_number\_13403SRS018888\_contig\_number\_1058SRS018984\_contig\_number\_5748SRS019030\_contig\_number\_16281SRS019068\_contig\_number\_68069SRS019178\_contig\_number\_5223SRS019267\_contig\_number\_2163SRS019397\_contig\_number\_1410SRS019601\_contig\_number\_23322SRS019787\_contig\_number\_4410SRS020394\_contig\_number\_13542SRS020622\_contig\_number\_4471SRS021219\_contig\_number\_contig-100\_118.67976SRS022071\_contig\_number\_14806SRS022713\_contig\_number\_4959SRS023526\_contig\_number\_27128SRS023715\_contig\_number\_25807SRS023829\_contig\_number\_335SRS023971\_contig\_number\_4779SRS043701\_contig\_number\_14543SRS043768\_contig\_number\_75SRS044535\_contig\_number\_16202SRS045195\_contig\_number\_1393SRS045645\_contig\_number\_29792SRS045713\_contig\_number\_20781SRS045826\_contig\_number\_22242SRS046369\_contig\_number\_4919SRS048164\_contig\_number\_24198SRS049712\_contig\_number\_10615SRS049773\_contig\_number\_contig-100\_1448.199362SRS050941\_contig\_number\_15083SRS051610\_contig\_number\_contig-100\_122.72973SRS052027\_contig\_number\_1115SRS052078\_contig\_number\_1731SRS053398\_contig\_number\_12317SRS054059\_contig\_number\_3826SRS054590\_contig\_number\_323SRS056259\_contig\_number\_28150SRS056273\_contig\_number\_23962SRS057478\_contig\_number\_5373SRS058145\_contig\_number\_1928SRS058770\_contig\_number\_15594SRS062701\_contig\_number\_5634SRS063040\_contig\_number\_22362SRS063370\_contig\_number\_12380SRS064645\_contig\_number\_231SRS065397\_contig\_number\_3362SRS065504\_contig\_number\_3395SRS074964\_contig\_number\_3879SRS075078\_contig\_number\_contig-100\_1765.176248SRS075398\_contig\_number\_853SRS075773\_contig\_number\_30616SRS075984\_contig\_number\_12638SRS076804\_contig\_number\_8610SRS076929\_contig\_number\_contig-100\_350.135616SRS077086\_contig\_number\_7274SRS077194\_contig\_number\_22252SRS077392\_contig\_number\_26522SRS077454\_contig\_number\_14634SRS077849\_contig\_number\_12603SRS098061\_contig\_number\_9197SRS098571\_contig\_number\_44810SRS100021\_contig\_number\_4360SRS104036\_contig\_number\_1145SRS1041036\_contig\_number\_1485SRS1041038\_contig\_number\_9420SRS1041039\_contig\_number\_10581SRS1041090\_contig\_number\_5309SRS1041092\_contig\_number\_4625SRS1041095\_contig\_number\_1969SRS1041129\_contig\_number\_1825SRS1041136\_contig\_number\_22701SRS1041137\_contig\_number\_29543SRS1041138\_contig\_number\_10844SRS1041142\_contig\_number\_8808SRS104311\_contig\_number\_15718SRS104327\_contig\_number\_2667SRS1055038\_contig\_number\_5348SRS1055049\_contig\_number\_7003SRS1055069\_contig\_number\_12233SRS1055076\_contig\_number\_3834SRS142503\_contig\_number\_54155SRS142712\_contig\_number\_4051SRS143342\_contig\_number\_17033SRS143523\_contig\_number\_3627SRS143895\_contig\_number\_contig-100\_1856.249117SRS144183\_contig\_number\_38073SRS144297\_contig\_number\_14515SRS144362\_contig\_number\_19827SRS144506\_contig\_number\_8378SRS145497\_contig\_number\_12606SRS146764\_contig\_number\_20366SRS146813\_contig\_number\_4376SRS147271\_contig\_number\_28083SRS147272\_contig\_number\_554SRS147377\_contig\_number\_644SRS147614\_contig\_number\_3215SRS148196\_contig\_number\_43268SRS148253\_contig\_number\_9593SRS148511\_contig\_number\_11511SRS148784\_contig\_number\_28923SRS148817\_contig\_number\_5042SRS148874\_contig\_number\_1330SRS149244\_contig\_number\_2241SRS893230\_contig\_number\_2747SRS893231\_contig\_number\_1658SRS893253\_contig\_number\_3008SRS893256\_contig\_number\_955SRS893259\_contig\_number\_1565SRS893285\_contig\_number\_2307SRS893288\_contig\_number\_465SRS893342\_contig\_number\_contig-100\_32.56208SRS893382\_contig\_number\_5681SRS971275\_contig\_number\_43058SRS971276\_contig\_number\_2680
143
species
823

658662
4
species

SRS017103\_contig\_number\_1926SRS053214\_contig\_number\_6071SRS077127\_contig\_number\_11190SRS077730\_contig\_number\_3171


SRS013951\_contig\_number\_37283SRS015431\_contig\_number\_22358SRS047014\_contig\_number\_21927SRS050299\_contig\_number\_10711SRS053649\_contig\_number\_11507SRS078419\_contig\_number\_36398SRS143876\_contig\_number\_37442SRS893252\_contig\_number\_17290
8
species
46503

2293117
species
1

SRS020328\_contig\_number\_14139

species
8

SRS016335\_contig\_number\_47861SRS017433\_contig\_number\_17558SRS019161\_contig\_number\_7120SRS049959\_contig\_number\_49056SRS075021\_contig\_number\_7745SRS076976\_contig\_number\_19752SRS142599\_contig\_number\_18779SRS142890\_contig\_number\_27794
563193

2293120
1
species

SRS150029\_contig\_number\_17779

469591

SRS1041091\_contig\_number\_15759
species
1

2293116
1
species

SRS1041133\_contig\_number\_25736

1262912
species
2

SRS050925\_contig\_number\_6252SRS104693\_contig\_number\_contig-100\_25.79858

species
3

SRS024388\_contig\_number\_11737SRS098717\_contig\_number\_1690SRS1041132\_contig\_number\_6105
2293114

815
14
family


SRS015890\_contig\_number\_8625SRS024435\_contig\_number\_3084SRS050752\_contig\_number\_30685SRS1041118\_contig\_number\_17589
816
genus
14
4

3
species

SRS020233\_contig\_number\_47220SRS058723\_contig\_number\_1567SRS064973\_contig\_number\_782
821

28116

SRS014459\_contig\_number\_15519SRS051882\_contig\_number\_4958
species
2

469589
1
species

SRS050026\_contig\_number\_9026

2292941
1
species

SRS893383\_contig\_number\_19975


SRS097920\_contig\_number\_15651SRS097958\_contig\_number\_contig-100\_270.135511
species
2
820

818
species
1

SRS142542\_contig\_number\_19598

171551
family
16

16
genus
836

species
16

SRS013800\_contig\_number\_19522SRS016954\_contig\_number\_13995SRS019445\_contig\_number\_contig-100\_1273.179601SRS019496\_contig\_number\_14491SRS022609\_contig\_number\_37833SRS024132\_contig\_number\_38519SRS043411\_contig\_number\_9266SRS078242\_contig\_number\_26451SRS098655\_contig\_number\_1526SRS1041134\_contig\_number\_14428SRS104975\_contig\_number\_24090SRS140492\_contig\_number\_27757SRS144603\_contig\_number\_5090SRS147346\_contig\_number\_9988SRS148721\_contig\_number\_51413SRS893341\_contig\_number\_3464
658663

family
3
171550

1
3
genus
239759

SRS015190\_contig\_number\_11955

1288121
species
1

SRS017701\_contig\_number\_1583

214856
1
species

SRS011084\_contig\_number\_3137
